# First-Trimester Ultrasound Imaging for Prenatal Assessment of the Extended Cardiovascular System Using the Cardiovascular System Sonographic Evaluation Algorithm (CASSEAL)

**DOI:** 10.3390/jcdd10080340

**Published:** 2023-08-07

**Authors:** Zita M. Gambacorti-Passerini, Cristina Martínez Payo, Coral Bravo Arribas, Santiago García-Tizón Larroca, Natalio García-Honduvilla, Miguel A. Ortega, Ricardo Pérez Fernández-Pachecho, Juan De León Luis

**Affiliations:** 1 Department of Obstetrics and Gynecology, University Hospital Gregorio Marañón, 28009 Madrid, Spain; zita.gambacorti@gmail.com (Z.M.G.-P.); cmartinezpy@gmail.com (C.M.P.); cbravoarribas@gmail.com (C.B.A.); gineteca@gmail.com (S.G.-T.L.); rperesf.hgugm@salud.madrid.org (R.P.F.-P.); jaleon@ucm.es (J.D.L.L.); 2 Health Research Institute Gregorio Marañón, 28009 Madrid, Spain; 3 Maternal and Infant Research Investigation Unit, Alonso Family Foundation (UDIMIFFA), 28009 Madrid, Spain; 4 Department of Public and Maternal and Child Health, School of Medicine, Complutense University of Madrid, 28040 Madrid, Spain; 5 Department of Medicine and Medical Specialities, Faculty of Medicine and Health Sciences, University of Alcalá, 28801 Alcalá de Henares, Spain; natalio.garcia@uah.es; 6 Ramón y Cajal Institute of Sanitary Research (IRYCIS), 28034 Madrid, Spain

**Keywords:** first trimester, extended cardiovascular system, sonographic protocol, congenital heart diseases, prenatal ultrasonic diagnosis

## Abstract

Introduction and objectives: To compare fetal images obtained at the first- and second-trimester ultrasound scan when applying the Cardiovascular System Sonographic Evaluation Algorithm (CASSEAL). Methods: Using the CASSEAL protocol, nine sequential axial views were acquired in B-mode and color Doppler at the first- and second-trimester ultrasound scans, visualizing the main components of the extended fetal cardiovascular system. Images were compared qualitatively between both trimesters. Results: We obtained comparable images for all the nine axial views described in the CASSEAL protocol, with few differences and limitations. Conclusions: The CASSEAL protocol is reproducible in the first trimester, and could help in the early detection of fetal cardiovascular abnormalities. It represents a promising additional tool in order to increase the CHD detection rate.

## 1. Introduction

Cardiovascular anomalies are the most common birth defect worldwide, with an estimated incidence that progressively increased over the past decades from 1/200 live births [1] to almost 1/100 live births [2]. Furthermore, in most cases it occurs in patients deemed to be at a low risk [3], thus representing a challenge for prenatal screening. Although most congenital heart diseases (CHDs) are minor, about half of these may be serious [4]. Importantly, prenatal diagnosis may reduce morbidity and mortality in these neonates [5,6].

The gold standard for prenatal CHD diagnosis in many countries involves second-trimester evaluation of cardiac anatomy, but great differences in detection rate (between 30–60% and 80–90%) and in how this screening is performed are reported [7,8]. In order to increase its detection rate, several protocols have been presented since the introduction of a fetal ultrasound scan. Moreover, in the last decades, with the inclusion of sonographic markers for aneuploidy in the screening for cardiac disease, greater attention has been shifted from the second to first-trimester echocardiography [9,10]. This increased interest in early prenatal diagnosis has been accompanied by a technical enhancement of the ultrasound machine and evidence on the safety of this practice [11].

As described in the second trimester, there is great difference in routine first-trimester screening for CHD between different centers, and little international consensus is available to guide clinicians on how to routinely perform first-trimester echocardiography [12,13,14].

It is well known that the cardiovascular system develops concomitantly with other adjacent systems and organs during the embryonic period, which may also be affected. Such areas include the heart, supra-aortic vessels, portal system and the thymus, thus composing the extended cardiovascular system. The Cardiovascular System Sonographic Evaluation Algorithm (CASSEAL) has been described in the second-trimester scan and has been applied in routinary ultrasound scans at our center since 2015. It combines the current recommendations for the screening of CHD with the assessment of these additional areas [15]. The application of this protocol to the first-trimester scan may improve the prenatal diagnosis of CHD by supporting clinicians in performing a systematic assessment of the extended cardiovascular system. The main benefit in the daily use of this tool is that it could contribute to the early detection of CHD or fetal vascular anomalies.

The aim of this work is to present the visualization of the same axial views described in the CASSEAL protocol in both the second and first trimesters.

## 2. Patients and Methods

We present a comparison between the nine transverse views of the algorithm acquired in the second trimester, and those acquired during the first-trimester scan (11–13 + 6 gestational weeks) in the context of a longitudinal observational study that we are carrying on at our hospital, after approval from our institutional ethical committee.

We followed the CASSEAL protocol to evaluate the cardiovascular system, which is the standard technique at our center in the second trimester of pregnancy (transabdominal 4–8 MHz and vaginal 4–9 MHz transducers, Samsung), but we applied it also in the first-trimester ultrasound study. The study was performed using the transabdominal transducer routinary, and in case of poor visualization the transvaginal transducer was used as well.

**Figure 1:**
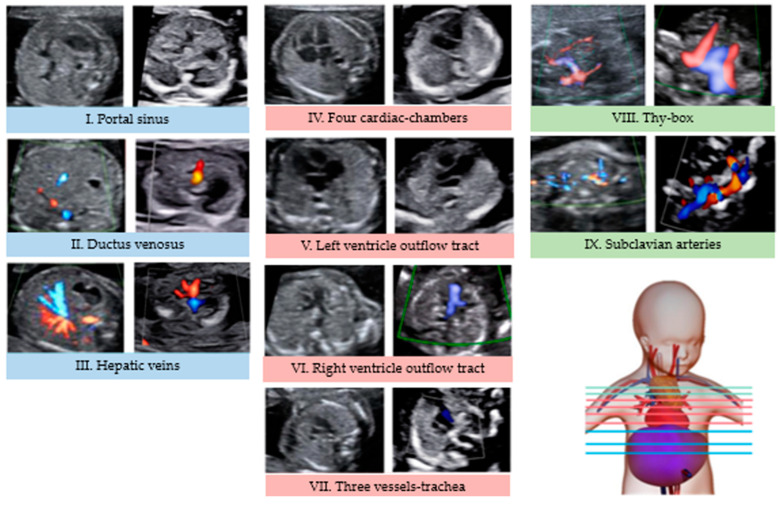
Axial views of fetal cardiovascular system at first and second trimester. Images on the left of each box were obtained from second trimester scan and on the right, from first trimester scan. Colored in blue images of the infracardiac territory; in red, images of the heart area and in green, images of the supracardiac territory.

Using the CASSEAL protocol, nine sequential axial views were acquired in B-mode, reproducing the CASSEAL protocol and visualizing the main components of the extended fetal cardiovascular system [15] as follows: I, portal sinus; II, ductus venosus (DV); III, hepatic veins (HV); IV, four cardiac chambers; V, left ventricle outflow tract; VI, right ventricle outflow tract; VII, three vessels and trachea (3VT); VIII, thy-box; and IX, subclavian arteries (Figure 1). In the case of limited visualization, color Doppler technique could be used.

The study protocol was approved by the local Ethics Committee with the code ALEESCA1T.

## 3. Results

We present a comparison of the images obtained at each trimester, to evaluate the feasibility and visualization of each axial view, including the anatomical landmarks of each axial view, as described in the CASSEAL protocol [15].

A set of nine axial images (Figure 1) was obtained for first and second trimesters. In these cases, no cardiovascular abnormality in the fetus was found with the CASSEAL assessment.

We describe the nine axial views obtained at each trimester as follows, highlighting the main differences between each pair of images.

### 3.1. Infracardiac Territory (I to III View)

In these first three pairs of images, we can see the reproducibility of the images obtained at each trimester in the area of the umbilico-portal vessels.

**Figure 2:**
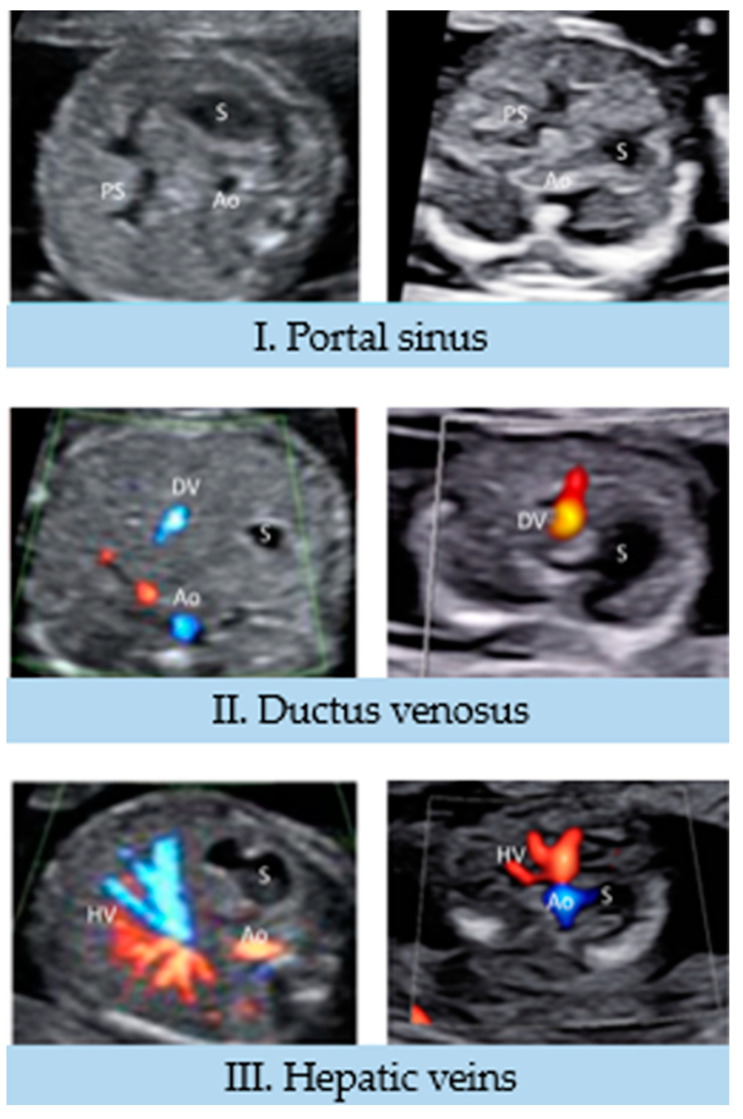
Infracardiac territory (I to III View). Ao, abdominal aorta; DV, ductus venosis; HV, hepatic veins; PS, portal sinus; S, stomach.

I View: this is an axial plane of the upper fetal abdomen at the level of the entrance of the umbilical vein (UV). Visible frames of reference are the following: the spine in the back, the descending aorta to the left, the inferior vena cava (IVC) anterior and to the right, the stomach in the left abdomen, and the portal sinus (PS) on the right side of the abdomen. In our image, obtained at first trimester, we could clearly identify the entrance of the UV, the PS on the right, the stomach and the spine, while it was more complicated to identify the descending aorta and the IVC. It was not possible to identify the left nor the right portal vein branches, while these details were visible in the second trimester study (Figure 2) [16].

II View: in this axial view, slightly cranial to I view, it is possible to identify the ductus venosus (DV), a thin vessel originating in the left portal vein and connecting this vessel with the IVC in a cranial, lateromedial, and anteroposterior direction. Other references of this plane are the stomach on the left, the spine in the back, and cross sections of the descending aorta and the IVC (Figure 2) [16]. Similarly to the previous view, we could not identify the IVC and descending aorta, while the other references were clearly visible.

III View: this is an axial plane of the fetal upper abdomen, above the DV, that allows for the visualization of the hepatic veins (HVs): right, middle and left. HVs converge with the DV in the IVC. Frames of the reference of this plane are the spine and the aorta at the back of the abdomen, and the stomach on the left (Figure 2) [16]. At the first trimester, we could clearly visualize the three branches of the HV, and the images obtained at both trimesters are well-correlated.

### 3.2. Heart Area (IV to VII Views)

**Figure 3:**
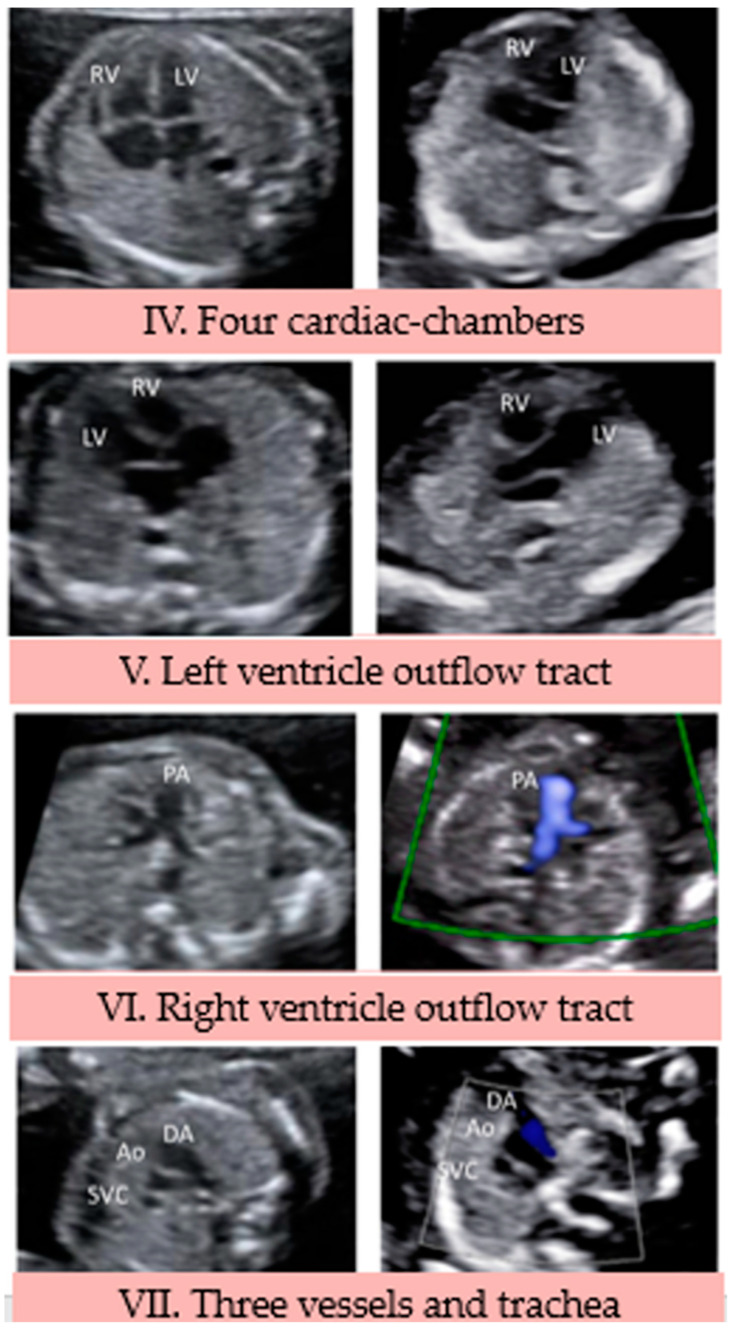
Heart area (IV to VII View). Ao, aorta; DA, ductus arteriosus, LV, left ventricle; PA, pulmonary artery; RV, right ventricle; SVC, superior vena cava.

IV View: this is a transverse view of the fetal thorax, showing the four-chamber view of the heart (Figure 3). Behind the left atrium the descending aorta can be seen. In our images we could identify those structures at both trimesters.

V View: angling the plane cranially and toward the right fetal side from the previous view, we can appreciate the outflow tract of the left ventricle and the aorta (Figure 2) [17]. We were able to identify those structures at both trimesters without the use of Doppler color.

VI View: if we follow slightly cranially and on the left fetal side from the previous view, we obtain a plane that shows the right ventricle outflow tract and the main pulmonary artery with its bifurcation into the right and left pulmonary arteries. The ascending and descending aorta and the superior vena cava (SVC) are also visible (Figure 3) [17]. In this plane we necessitated the use of color Doppler to clearly identify the pulmonary artery and its bifurcation.

VII View: moving toward the fetal head, a view of the three vessels and trachea is achieved (3VT). It shows, from left to right, the pulmonary artery connecting with the ductus arteriosus (DA), a transverse section of the aortic arch, the SVC and the trachea on the right (Figure 3). The aorta and the DA form a V shape with its vertex posteriorly and to the left of the trachea [18].

### 3.3. Supracardiac Territory (VIII and IX View)

**Figure 4:**
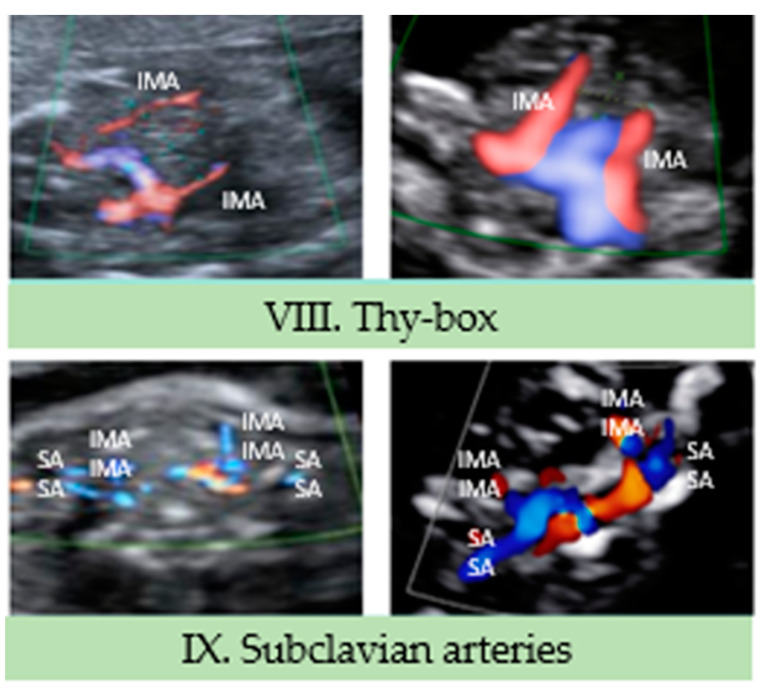
Supracardiac territory (VIII to IX View). IMA, internal mammary artery; SA, subclavian artery.

VIII View: in this thoracic transverse section, we can identify the fetal thymus, just cranially to the previous view (VII). It is possible to identify the thymus inside the thy-box, limited by the internal mammary arteries (laterally), the sternum (anteriorly) and the origin of the great vessels (posteriorly) (Figure 4) [19].

In the first trimester study, the mammary arteries appear to be less defined than in the second trimester study. However, they could be clearly identified with the use of color Doppler, thus allowing for a correct visualization of the anatomical limits of the fetal thymus.

IX View: the subclavian arteries may be seen in this transverse plane, with the typical “s-shape”, running toward the fetal arms and anteriorly to the trachea. As in the previous view, the subclavian arteries could be appropriately identified with the use of color Doppler.

## 4. Discussion

Our aim is to reproduce the CASSEAL protocol views in the first trimester and compare these images with those obtained in the second trimester, presented in a previous work [15] in order to evaluate its feasibility. As we previously stated, the CASSEAL protocol represents our standard technique for cardiovascular anomaly screening in the second trimester of pregnancy, and we are actually performing a longitudinal observational study in which the CASSEAL algorithm is also applied in the first-trimester ultrasound scan.

It is described in literature that imaging protocols increase screening performance, in terms of the higher detection rate of cardiac anomalies [15,20], even in the first trimester (11–14 weeks), highlighting the importance of well-described, feasible and reproducible imaging protocol for the first-trimester scan. This improvement is more evident in studies using outflow–tract views and color Doppler imaging, with a “dose-response” improvement in the detection rate with increasing detail of the anatomical study protocol [14]. As described in the review of Karim et al., studies using the most extensive anatomical protocol achieved detection rates in non-high-risk population that were comparable with those in the high-risk population [14].

The CASSEAL protocol includes not only these views and the use of color Doppler, but also other complementary views of the extended cardiovascular system, as previously described, thus representing a promising additional tool in order to increase the CHD detection rate. Increasing evidence shows the safety of using Doppler between 11 and 13 + 6 gestational weeks, while respecting the ALARA (as low as reasonably achievable) principle and maintaining the Thermal Index (TI) and the Mechanical Index (MI) below the limits indicated. Its use may be justified for clinical indications, as for the screening of aneuploidies and congenital heart disease [21].

In the images presented in this article, we show the reproducibility of the CASSEAL protocol in the first trimester, obtaining a good quality ultrasound image for each view. For some views, the images are somehow less defined, especially in the case of small vessel visualization (like the mammary or the subclavian arteries), but this does not prevent a correct identification of the key structures, thus allowing for identifying the normality or abnormality of the anatomical structures.

We know that there are some characteristics that will make this study intrinsically more challenging in the first compared to the second trimester: first of all, the fetus’ dimensions. We know that the mean abdominal circumference (percentile 50) at 11 and 14 gestational weeks is 45.4 mm and 80.6 mm, respectively, while it is 136.7 and 169.6 mm at 19 and 22 weeks [22,23]. The same happens for the thoracic circumference, which is reported to be 74.3 mm at 13 gestational weeks, while it is 125.9 and 154 mm at 19 and 22 weeks [24,25]. This will probably lead to a longer learning curve for the sonographers involved in the first-trimester scan, an appropriate allocation of time and the use of high-resolution ultrasound equipment, in order to a achieve higher detection rate. The medium time to complete the CASSEAL protocol in first trimester has not been strictly evaluated yet. We can state, however, that in the second trimester, the complete assessment takes 5.6 min (SD 4.2 min) by our group [15]. In the first trimester, we can assume a little bit more, at least until the learning curve is achieved, which could the situated around 30 evaluations.

Despite these inconvenience, if we consider the potential benefit of an early detection of fetal cardiac abnormalities, or at least the identification of a group of fetuses supposed to be at a high risk of CHD, those limitations could be solved by appropriate training and implementation of the general setting of the first trimester visit. An early suspicion of a CHD or vascular abnormality may allow for a second ultrasound assessment to confirm the finding, even by a more expert sonographer. Once the anomaly is confirmed, we can offer the patients early counseling, including invasive testing when needed and the chance of an early termination of pregnancy if this is their choice. Getting ahead of the second trimester morphology scan brings advantages in the matter of time for additional testing, information to the parents and decision-making process.

Further studies on the application of the CASSEAL protocol in routinary first-trimester visit, would provide interesting information on the feasibility and reproducibility of the protocol in clinical practice.

## 5. Conclusions

The application of the CASSEAL protocol to the first-trimester scan may improve the prenatal diagnosis of CHD. In this technical note, we show the reproducibility of the CASSEAL protocol in the first trimester, obtaining a good quality ultrasound image for each view. For some views, the images are somehow less defined, especially in the case of small vessels visualization (like the mammary or the subclavian arteries), but this does not prevent a correct identification of the key structures.

## Data Availability

The data used to support the findings of the present study are available from the corresponding author upon request.
